# Supplementary material for: Genome-wide analyses across Viridiplantae reveal the origin and diversification of small RNA pathway-related genes
Source: Commun Biol. 2021 Mar 25;4:412. doi: 10.1038/s42003-021-01933-5 (PMC7994812; doi:10.1038/s42003-021-01933-5)
Supplement: Supplementary file 2 — Supplementary Information [file 42003_2021_1933_MOESM2_ESM.pdf]

Supplementary Information

Genome-wide analyses across Viridiplantae reveal the origin and diversification of small RNA pathway-related genes

Sibo Wang[1,2#], Hongping Liang[1,3#], Yan Xu[1,3], Linzhou Li[1,4], Hongli Wang[1,3], Durgesh Nandini Sahu[1], Morten Petersen[2], Michael Melkonian[5], Sunil Kumar Sahu[1*] and Huan Liu[1,2*]

[1]State Key Laboratory of Agricultural Genomics, BGI-Shenzhen, Shenzhen 518083, China
[2]Department of Biology, University of Copenhagen, Copenhagen, Denmark
[3]BGI Education Center, University of Chinese Academy of Sciences, Shenzhen, China
[4]Department of Biotechnology and Biomedicine, Technical University of Denmark, Lyngby, Denmark
[5]Integrative Bioinformatics, Department Plant Microbe Interactions, Max Planck Institute for Plant Breeding Research, 50829 Cologne, Germany
#These authors contributed equally

*Correspondence address
Huan Liu, BGI-Shenzhen, Beishan Industrial Zone, Yantian District, Shenzhen 518083, China; Tel: +86 18025460332; E-mail: liuhuan@genomics.cn; ORCID: 0000-0003-3909-0931

Sunil Kumar Sahu, State Key Laboratory of Agricultural Genomics, BGI-Shenzhen, Shenzhen 518083, China; Tel: +86 13535124942; E-mail: sunilkumarsahu@genomics.cn; ORCID: 0000-0002-4742-9870

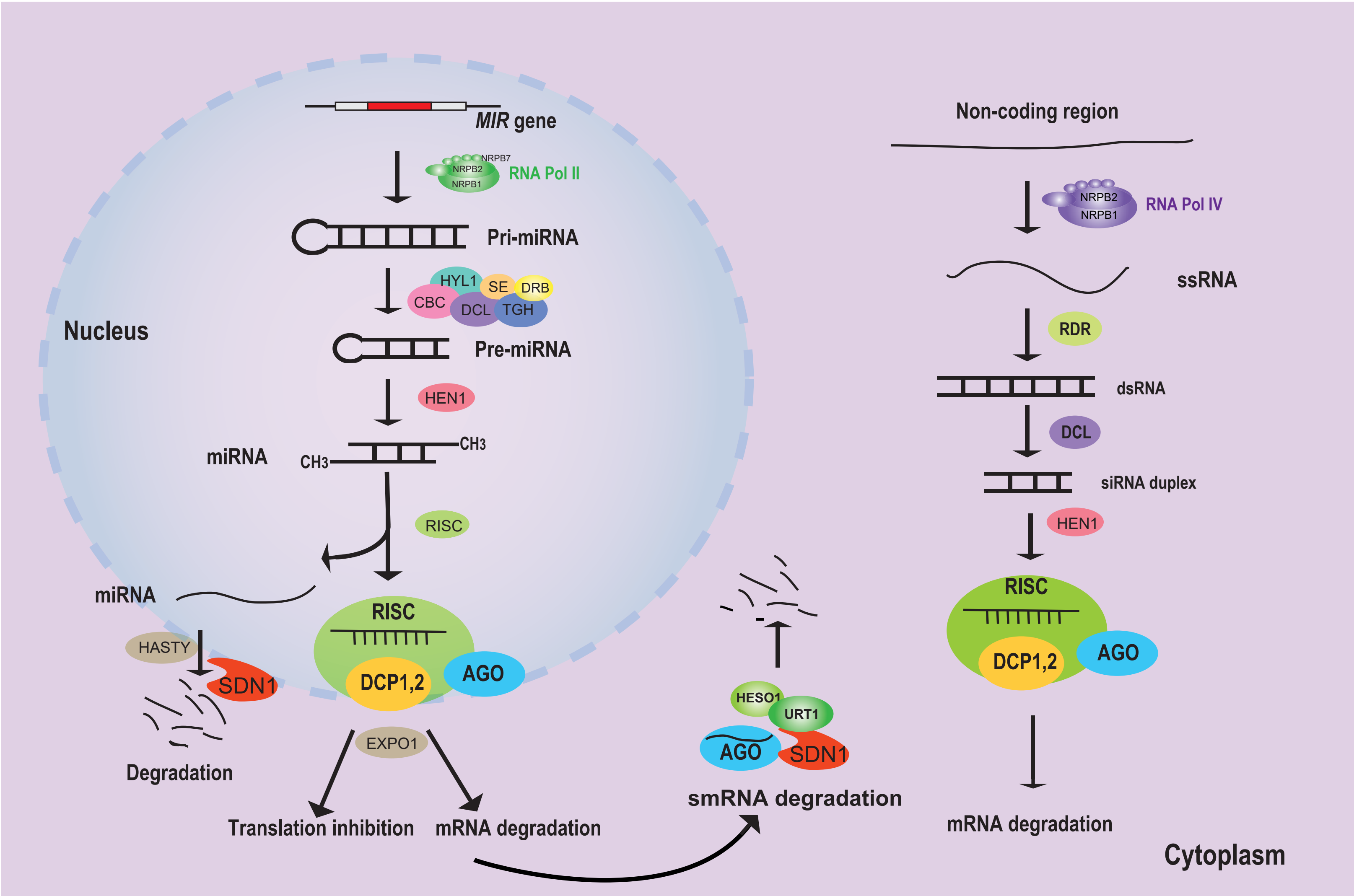

Figure S1. Schematic representation of the sRNA (miRNA and siRNA) biogenesis and degradation pathways
AGO, Protein argonaute; RNA Pol, RNA polymerase; CBC, Cap-binding complex; DCL, Dicer-like protein; DCP, de-capping protein; HEN1, Hua Enhancer 1; RDRs, RNA-Dependent RNA polymerases; RISC, RNA-induced silencing complex; SE, zinc-finger protein Serrate, TGH, Tough (a known miRNA processing regulator); HYL1, RNA binding protein hyponastic leaves; SE, zinc-finger protein SERRATE; DRB, double-stranded RNA binding; CBC, Cap Binding Complex; RISC, RNA-induced silencing complex; DCP, Decapping protein; EXPO1, Exportin 1; SDN1: small RNA degrading nuclease; URT1, RNA Uridylyltransferase1; HESO1: HEN1 suppressor1.

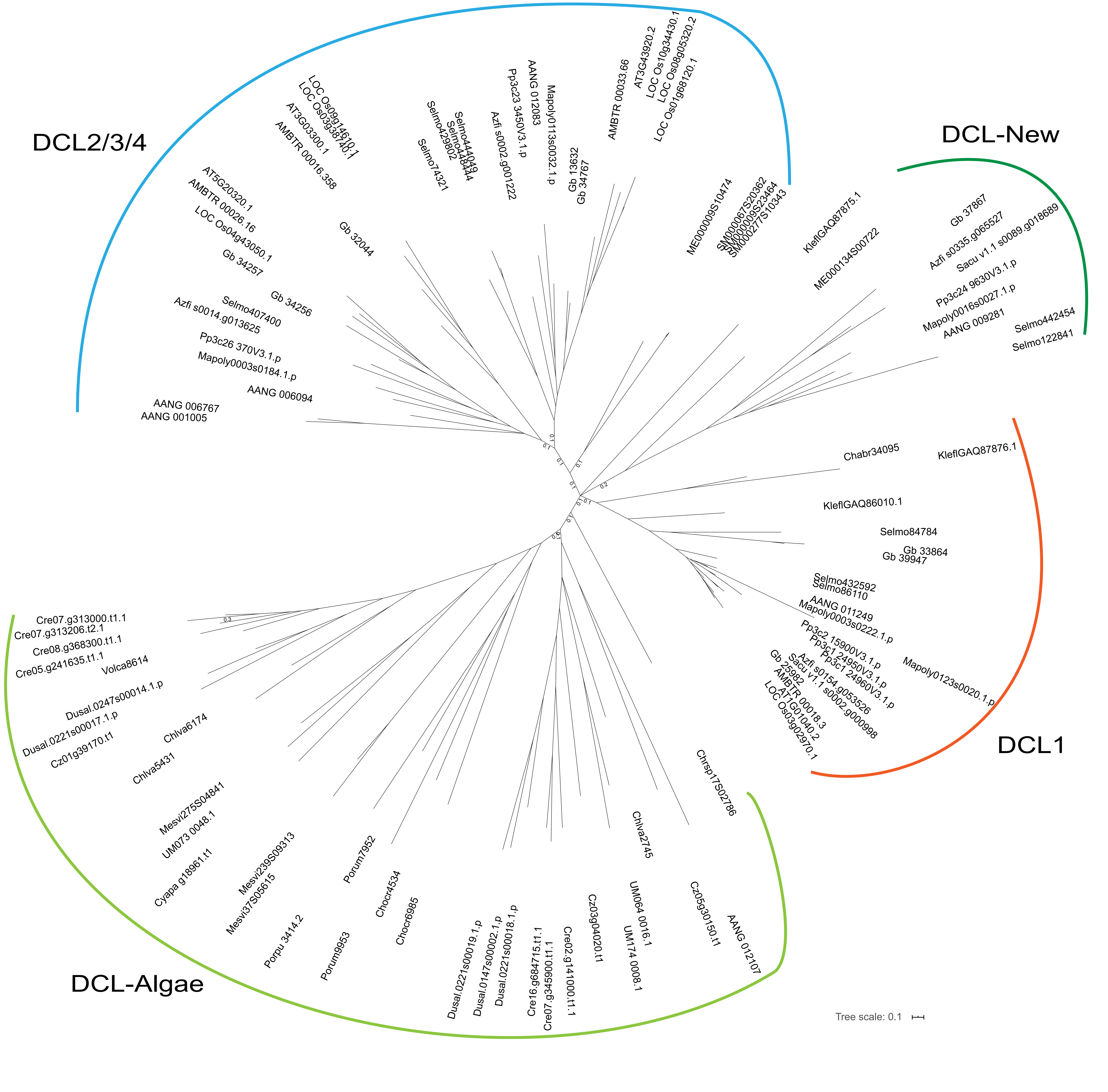

Fig. S2 The phylogenetic tree of DCL based on the Bayesian method

A Bayesian tree of DCL1 with 3 additional Zygnematophyceae from the 1KP dataset. The Bayesian tree was constructed by MrBayes (version 3.2.6) using the GTR-GAMMA evolutionary model with six Markov chains until the average standard deviations of split frequencies were less than 0.05 (500,000 generations). For species designations of sequence IDs see Supplementary data S3.

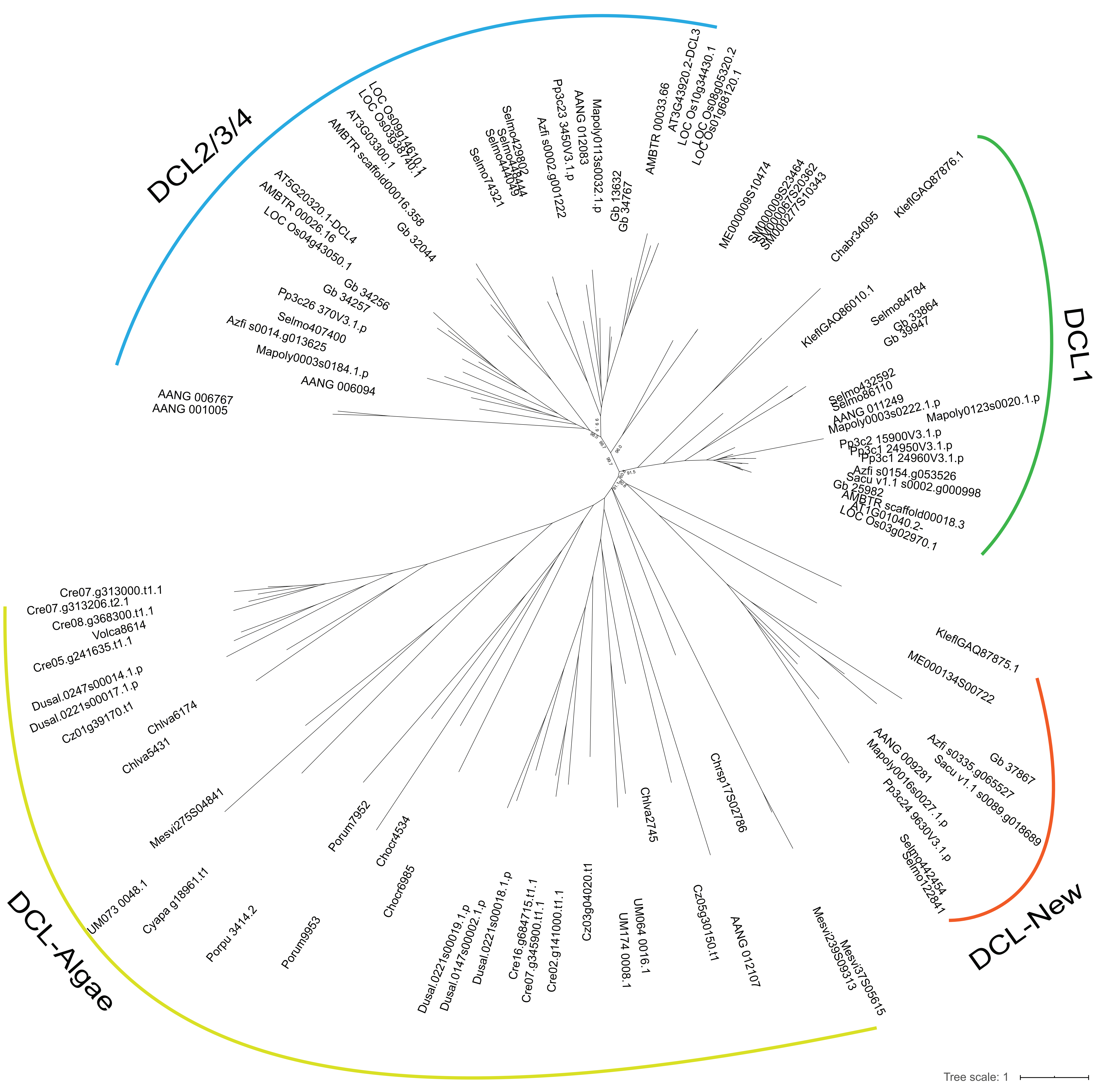

Fig. S3 The phylogenetic tree of DCL based on the IQ tree software

The IQ tree of DCL1 with 3 additional Zygnematophyceae. The multiple sequence alignments were processed by using MAFFT, and then IQtree software was used to generate the tree with the best model (LG+F+R7) by automatic predistortions.

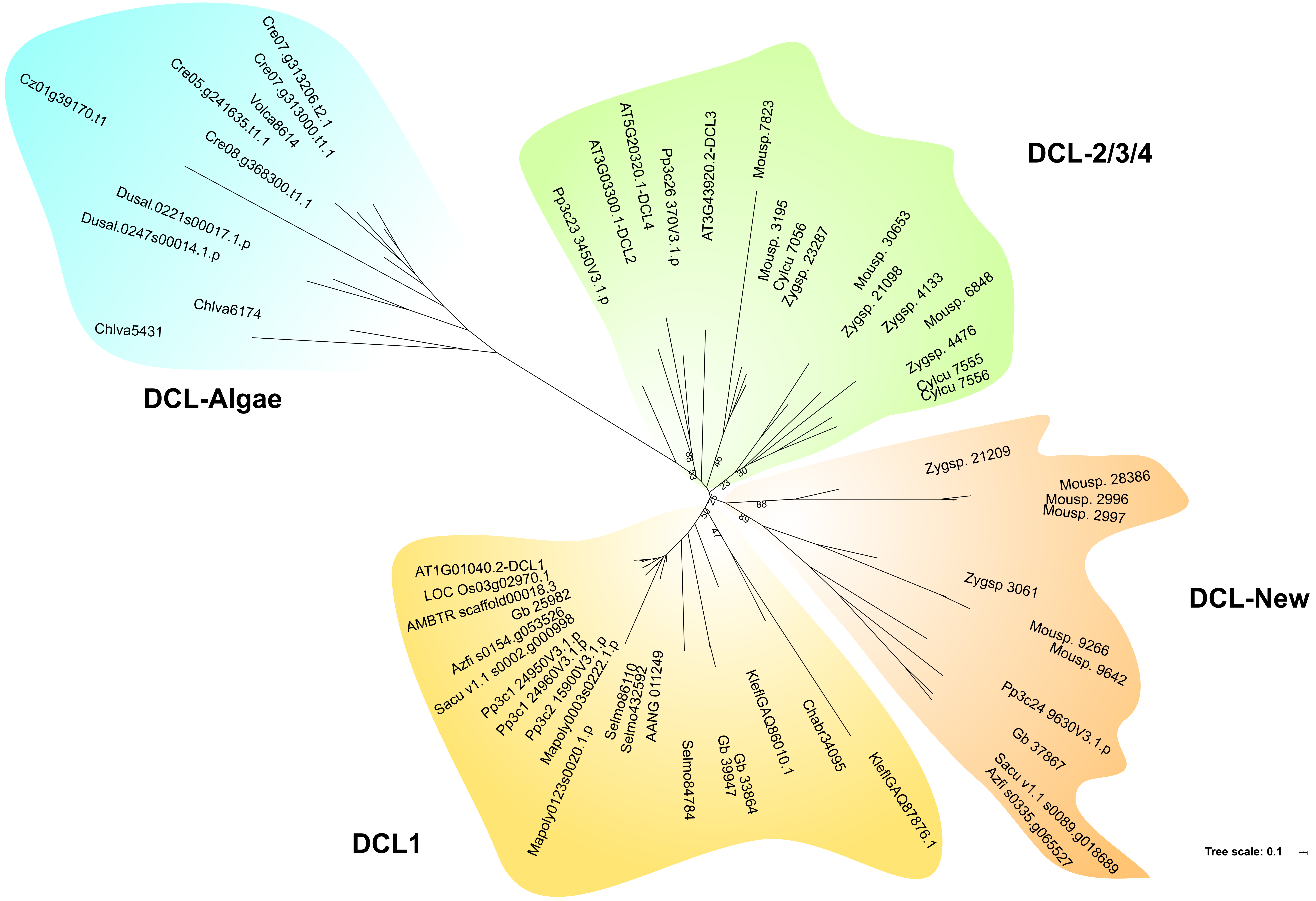

FigS4. The phylogenetic tree of DCL1 gene in Viridiplantae

A Maximum-likelihood tree of DCL1 with 3 additional Zygnematophyceae from the 1KP dataset. A detailed DCL phylogenetic tree is shown in Figure 2a. Only bootstrap values of the main clades are shown (0-100). The support for internal branch was assessed using 500 bootstrap replicates. The ID to corresponding species name is described in the Supplementary data S2.

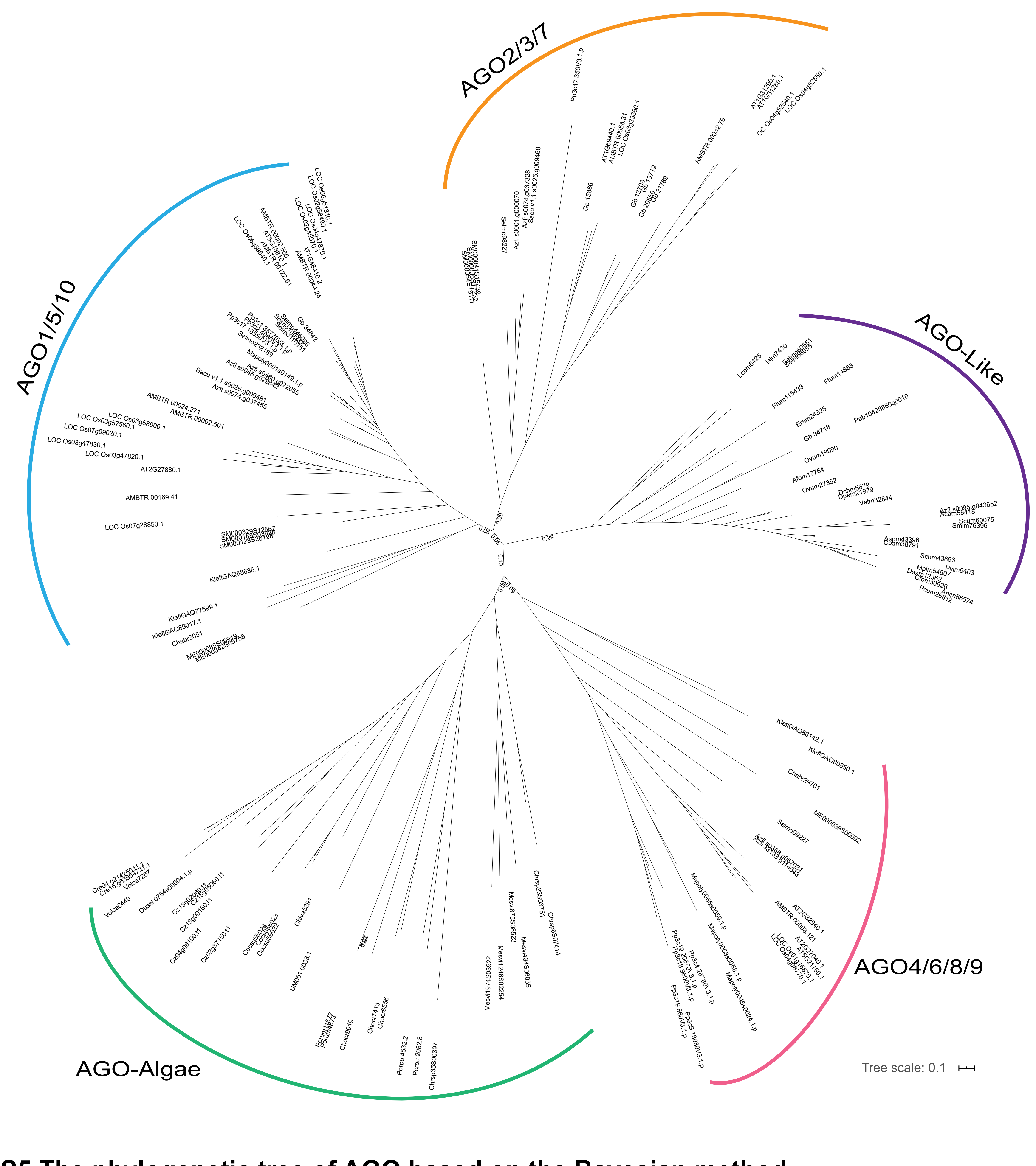

Fig. S5 The phylogenetic tree of AGO based on the Bayesian method

A Bayesian tree with additional transcriptome data of streptophyte algae from the 1KP dataset. The tree was constructed by MrBayes (version 3.2.6) using the GTR-GAMMA evolutionary model with six Markov chains until the average standard deviation of split frequencies were lesser than 0.05 (600,000 generations). For species designations of sequence IDs see Supplementary data S3.

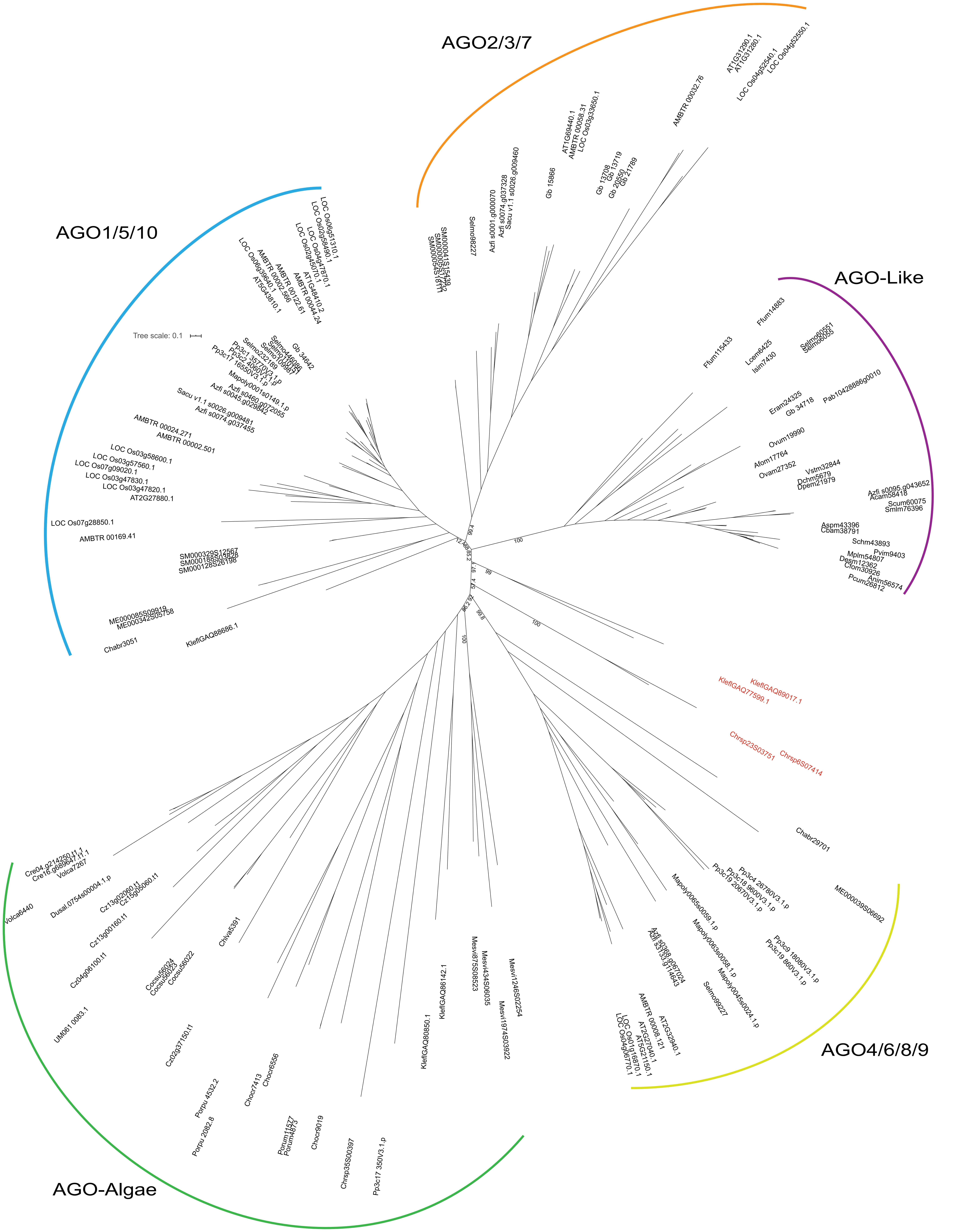

Fig. S6 The phylogenetic tree of AGO based on the IQ tree software

The IQ tree of AGO with additional transcriptome data of streptophyte algae from the 1KP dataset. The multiple sequence alignments were processed by using MAFFT, and then IQtree software was used to generate the tree with the best model (LG+F+R8) by automatic predistortions. For species designations of sequence IDs see Supplementary data S3.

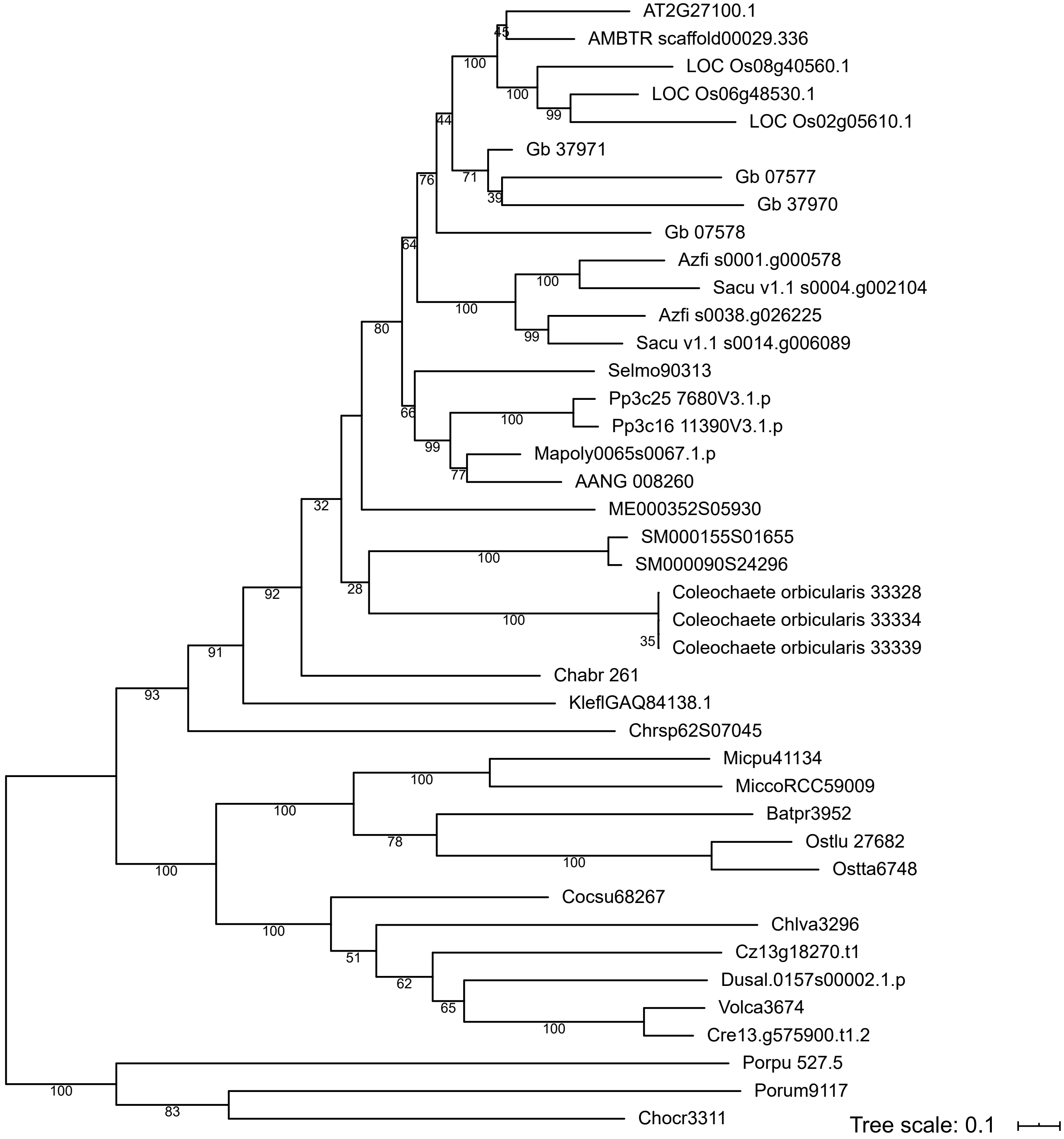

FigS7. The phylogenetic tree of the SE gene in Viridiplantae

A Maximum-likelihood tree of Serrate (SE) with 500 bootstrap replicates by RAxML with PROTCATGTR model. The tree is rooted in red algae with bootstrap values shown on each branch (0-100). The accession numbers of the sequences are tabulated in Supplementary data S3

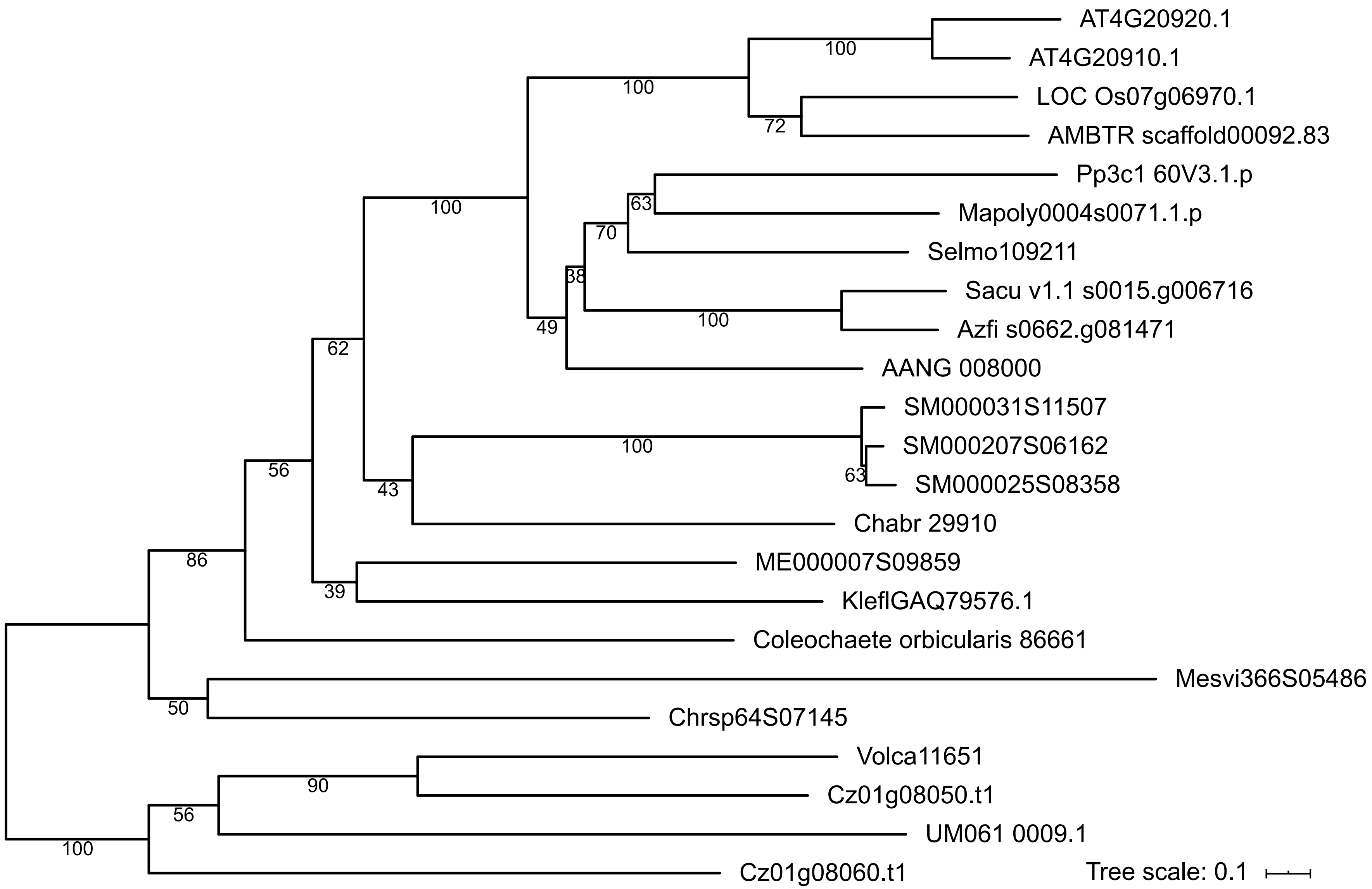

FigS8. The phylogenetic tree of the HEN1 gene in Viridiplantae

A Maximum-likelihood tree of HEN1 with 500 bootstrap replicates (PROTCATGTR model) by RAxML is shown here. The bootstrap values are shown on each branch (0-100). The accession numbers to the sequences are provided in Supplementary data S3.

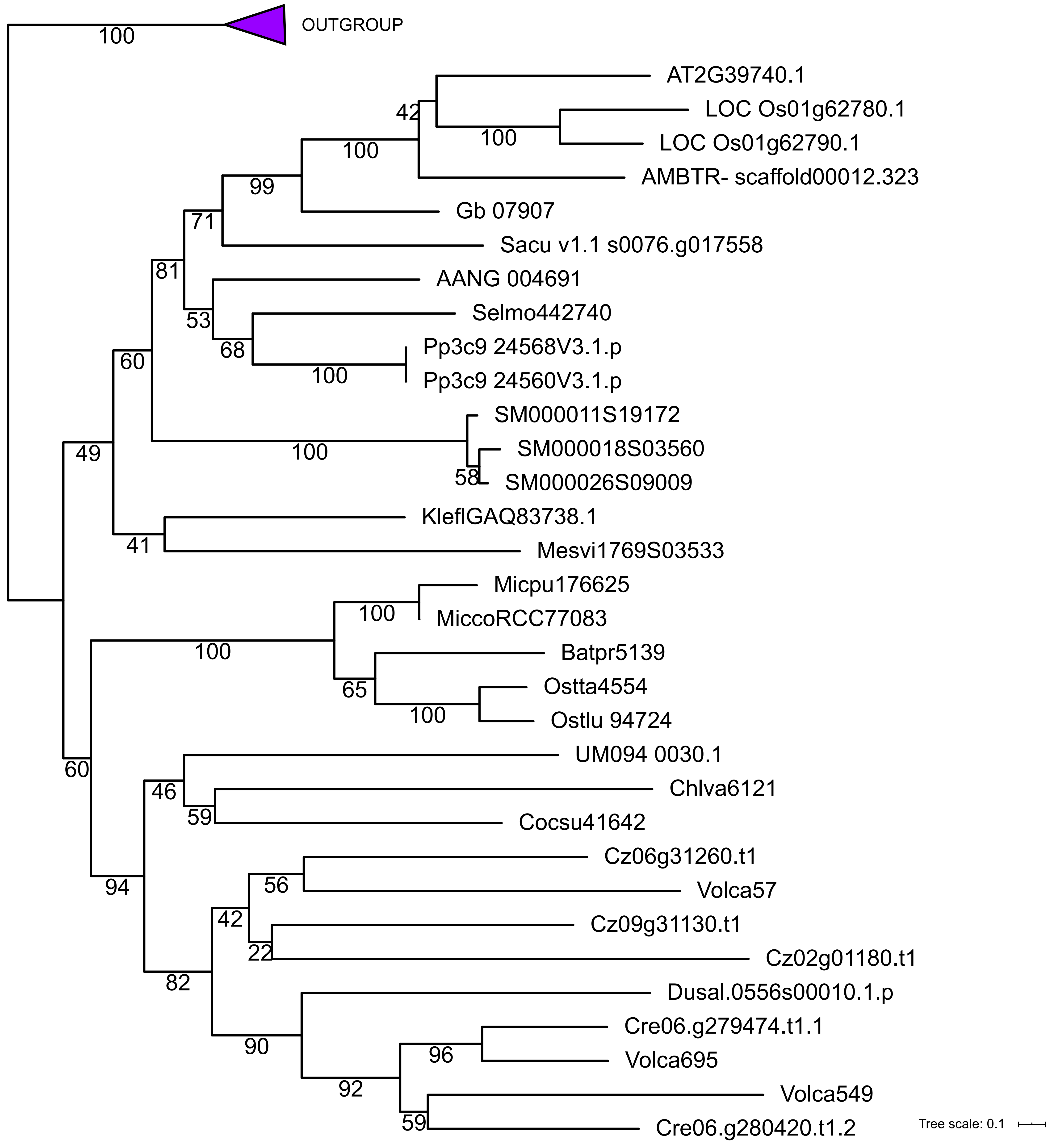

FigS9. The phylogenetic tree of the HESO1 gene in Viridiplantae
A Maximum-likelihood tree of HESO1 with 500 bootstrap replicates generated by RAxML with PROTCATGTR model, with HESO1 homologs AT3G15080 (Nucleotidyltransferase family protein) as an outgroup. The bootstrap values are shown on each branch (0-100) and accession numbers to the sequences can be found in Supplementary data S3.

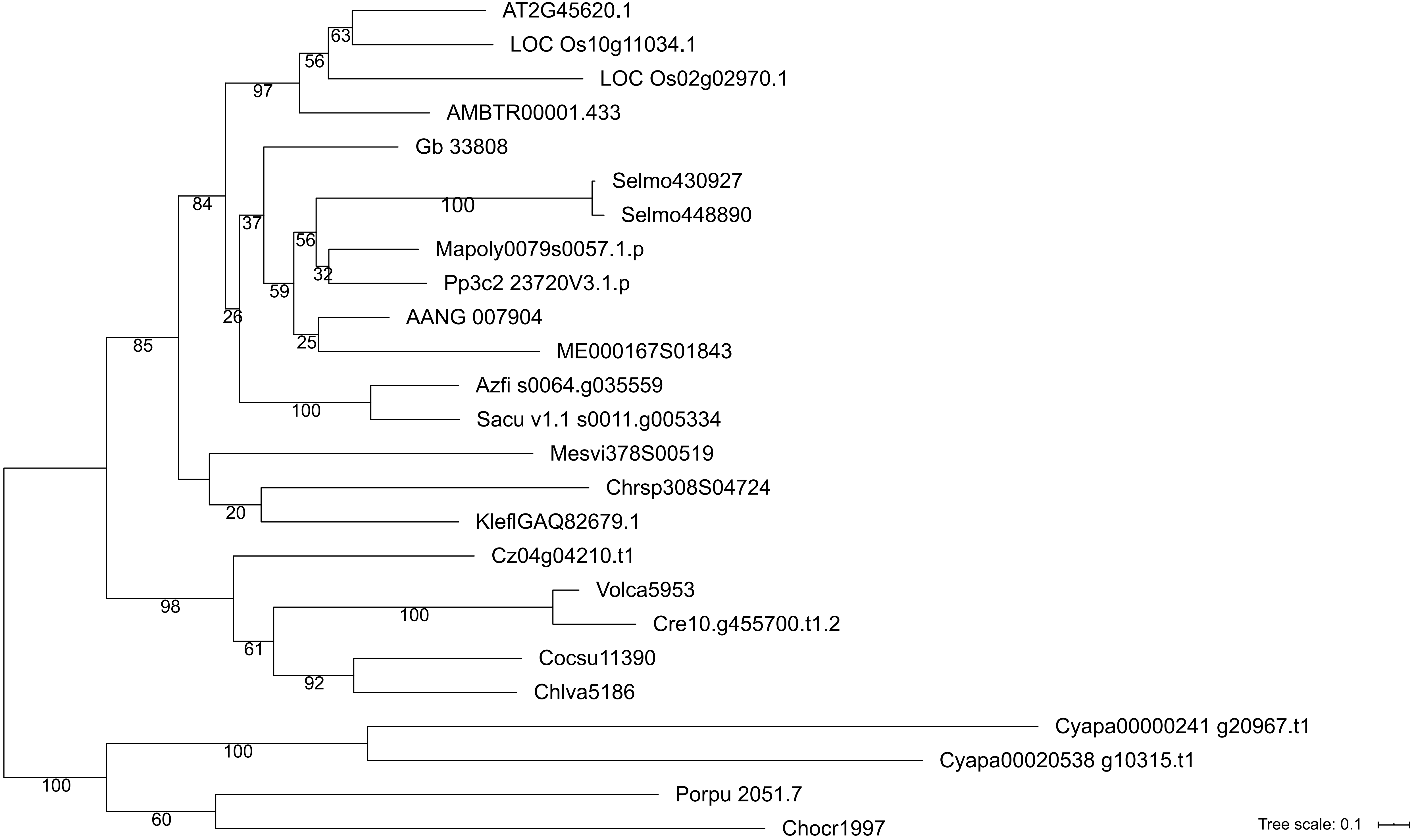

FigS10. The phylogenetic tree of the UTR1 gene in Viridiplantae

A Maximum-likelihood tree of UTR1 with 500 bootstrap replicates generated by RAxML with PROTCATGTR model. The tree is rooted with sequences from Rhodoplantae and Glaucoplantae. Bootstrap values are shown on each branch (0-100) and accession numbers to the sequences can be found in Supplementary data S3.

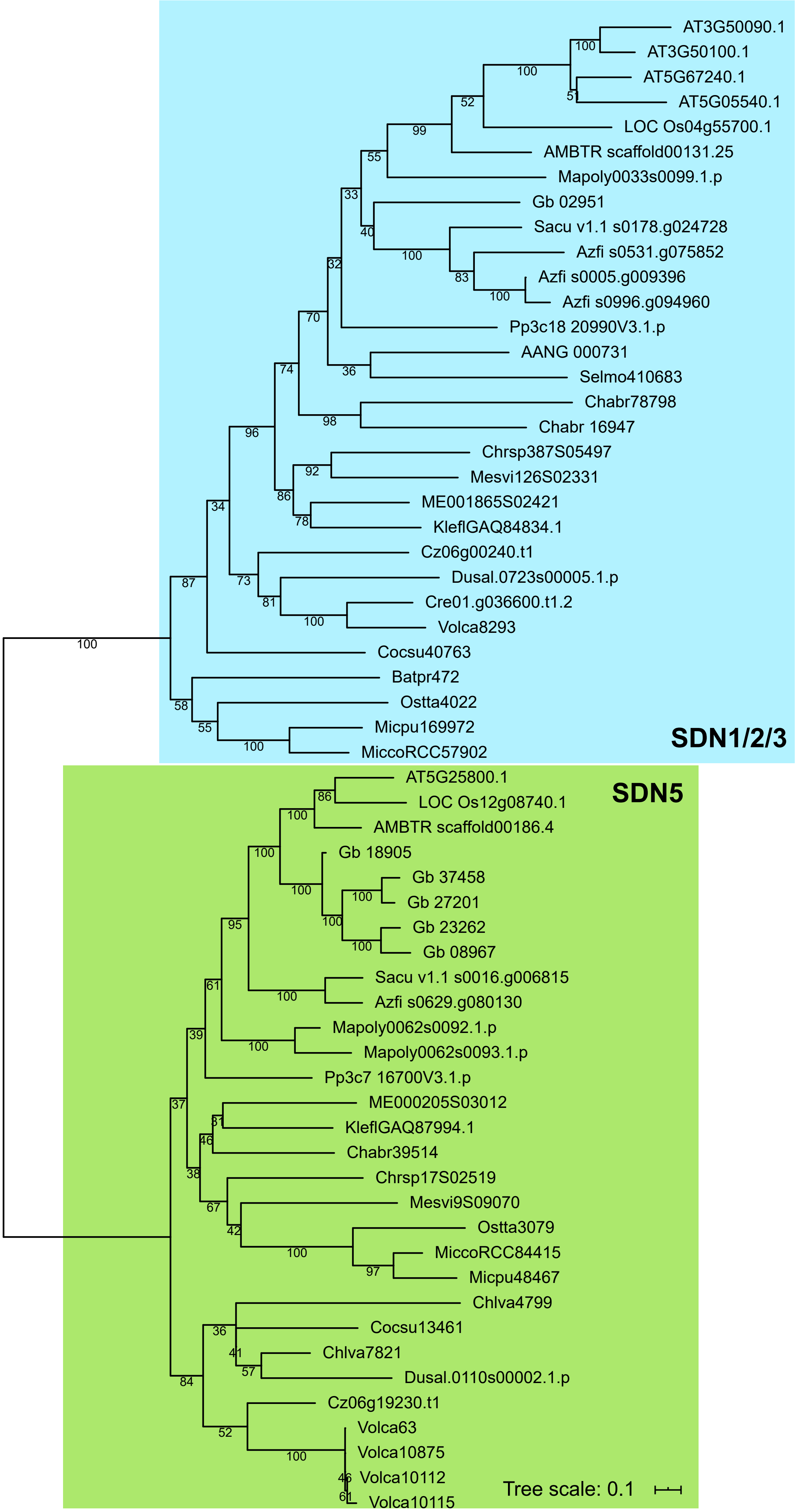

FigS11. The phylogenetic tree of the SDN gene in Viridiplantae
A Maximum-likelihood tree of SND1-5 with 500 bootstrap replicates generated by RAxML with PROTCATGTR model. Blue SND1/2/3/4, green SDN1.

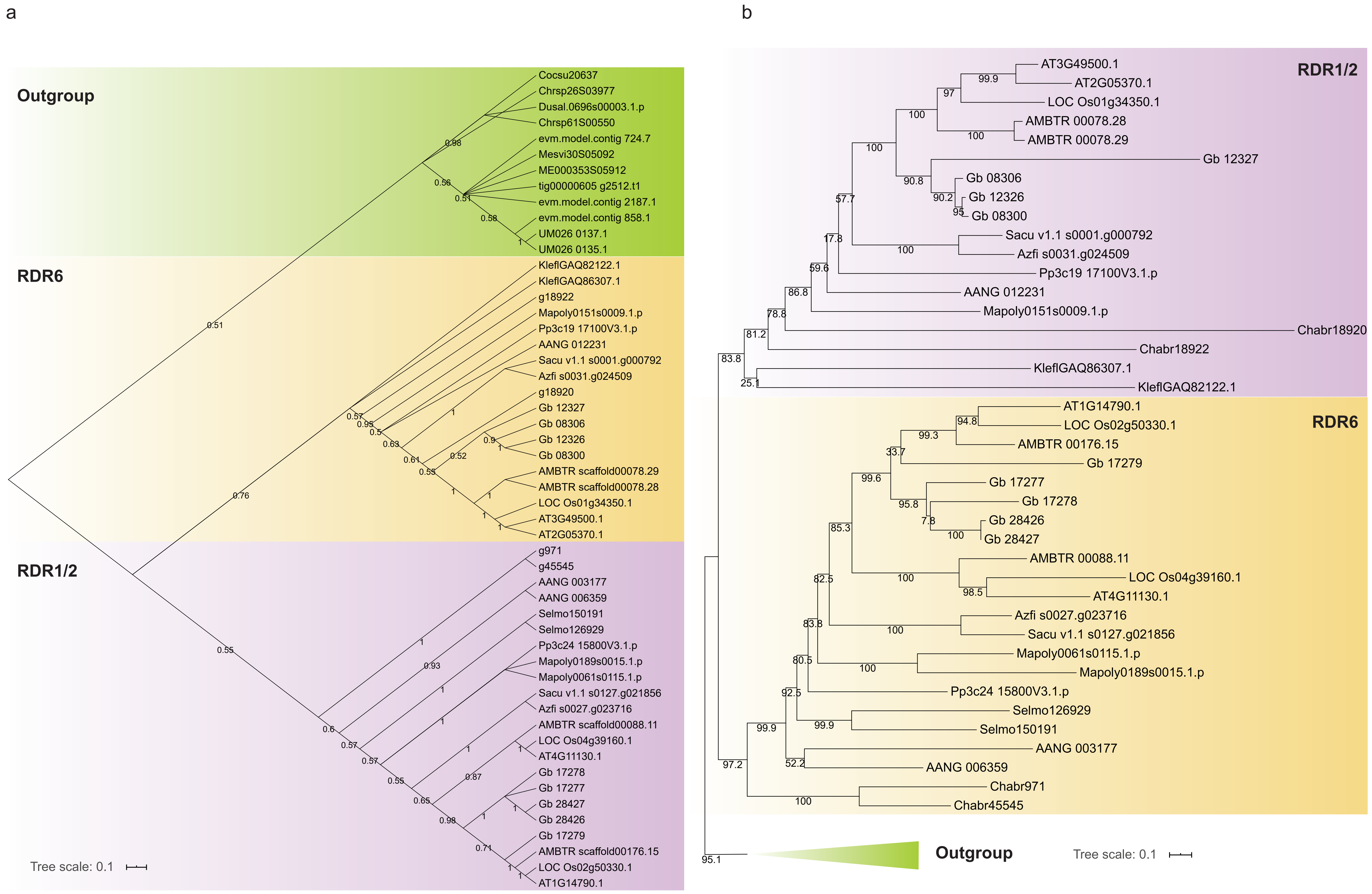

FigS12. The phylogenetic tree of RDR based on Bayes and IQ tree

(a) A Bayesian tree with additional transcriptome data of streptophyte algae from the 1KP dataset. The tree was constructed by MrBayes (version 3.2.6) using the GTR-GAMMA evolutionary model with six Markov chains until the average standard deviation of split frequencies were lesser than 0.05 (600,000 generations). For species designations of sequence IDs see Supplementary data S3. (b) The IQ tree of RDR with additional transcriptome data of streptophyte algae from the 1KP dataset. The multiple sequence alignments were processed by using MAFFT, and then IQtree software was used to generate the tree with the best model (LG+F+R8) by automatic predistortions. For species designations of sequence IDs see Supplementary data S3.

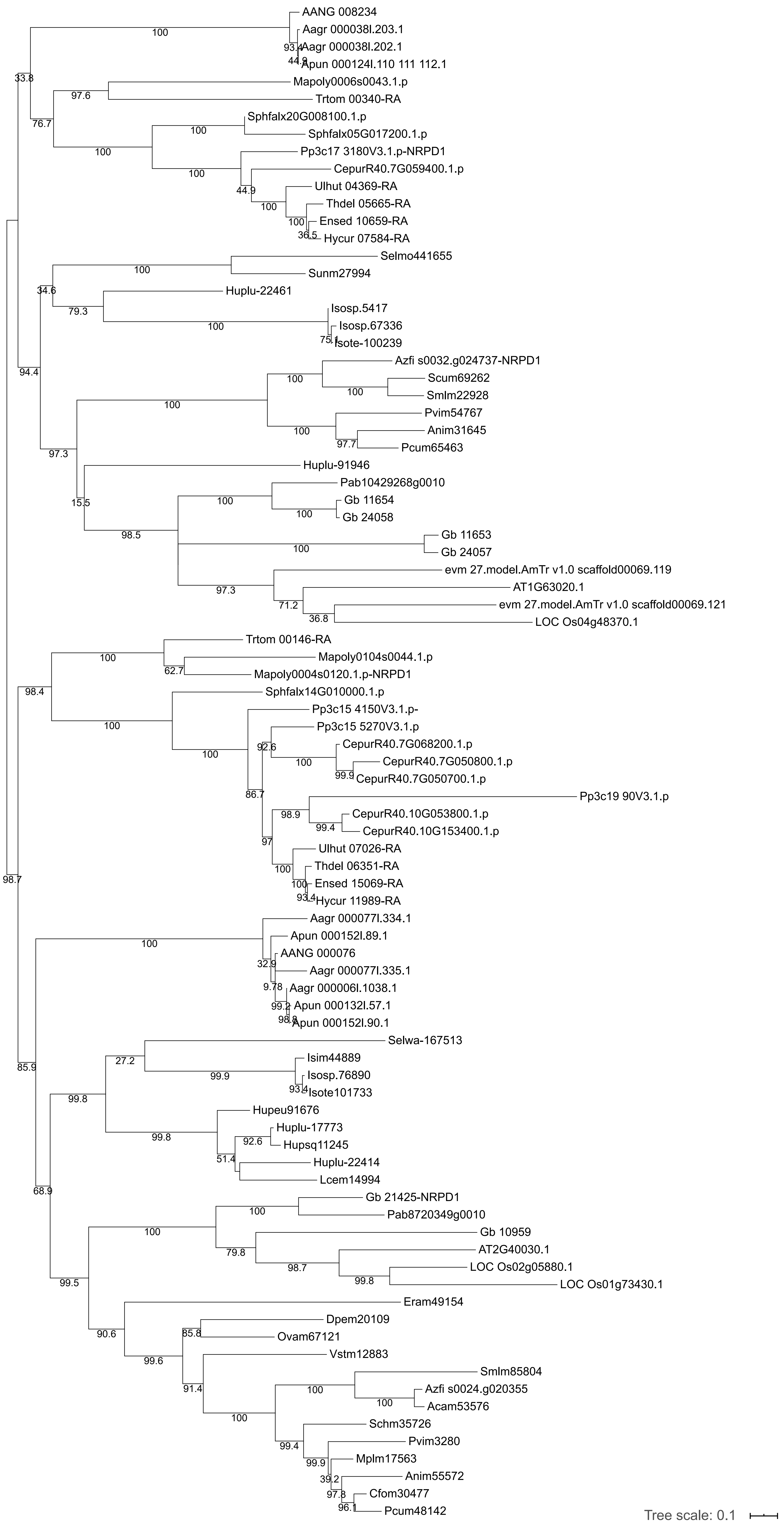

FigS13. The phylogenetic tree of subunit 1 of Pol IV and Pol V based on IQ tree

The IQ tree of NRPD1/E1 (subunit 1 of Pol IV and Pol V) with additional transcriptome data of streptophyte algae. The multiple sequence alignments were processed by using MAFFT, and then IQtree software was used to generate the tree with the best model (LG+F+R8) by automatic predistortions. For species designations of sequence IDs see Supplementary data S3.

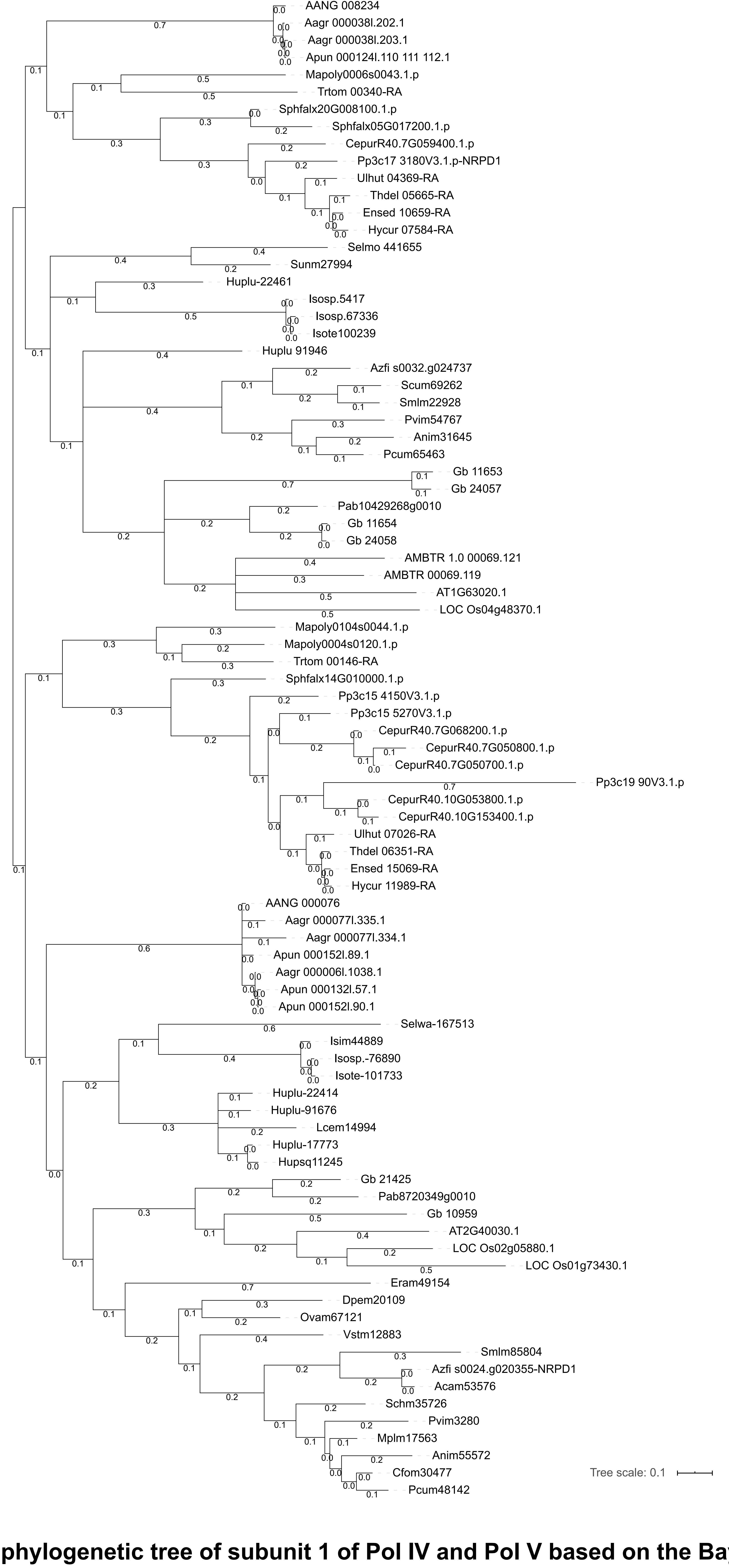

Fig. S14 The phylogenetic tree of subunit 1 of Pol IV and Pol V based on the Bayesian method
A Bayesian tree with additional transcriptome data of streptophyte algae. The tree was constructed by MrBayes (version 3.2.6) using the GTR-GAMMA evolutionary model with six Markov chains with 600,000 generations. For species designations of sequence IDs see Supplementary data S3.

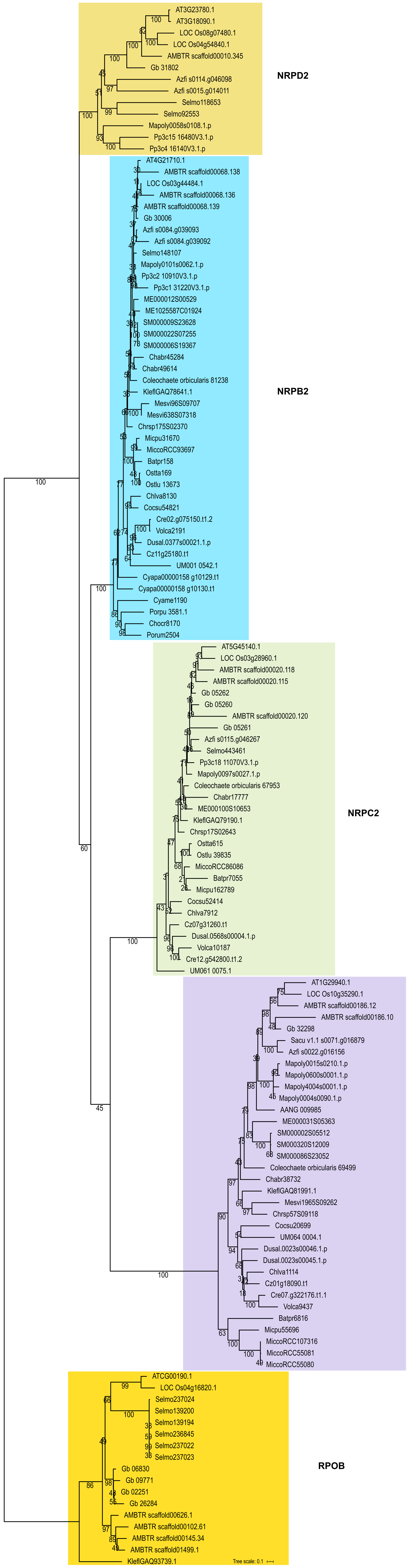

FigS15. The phylogenetic tree of subunit 2 of Pol I, Pol II, Pol III, Pol IV, and Pol V

A Maximum-likelihood tree of NRPA2/C2/B2/D2 with 500 bootstrap replicates generated by RAxML with PROTCATGTR model, with RPOB homologs as an outgroup. The bootstrap values are shown on each branch (0-100) and accession numbers to the sequences can be found in Supplementary data S3.

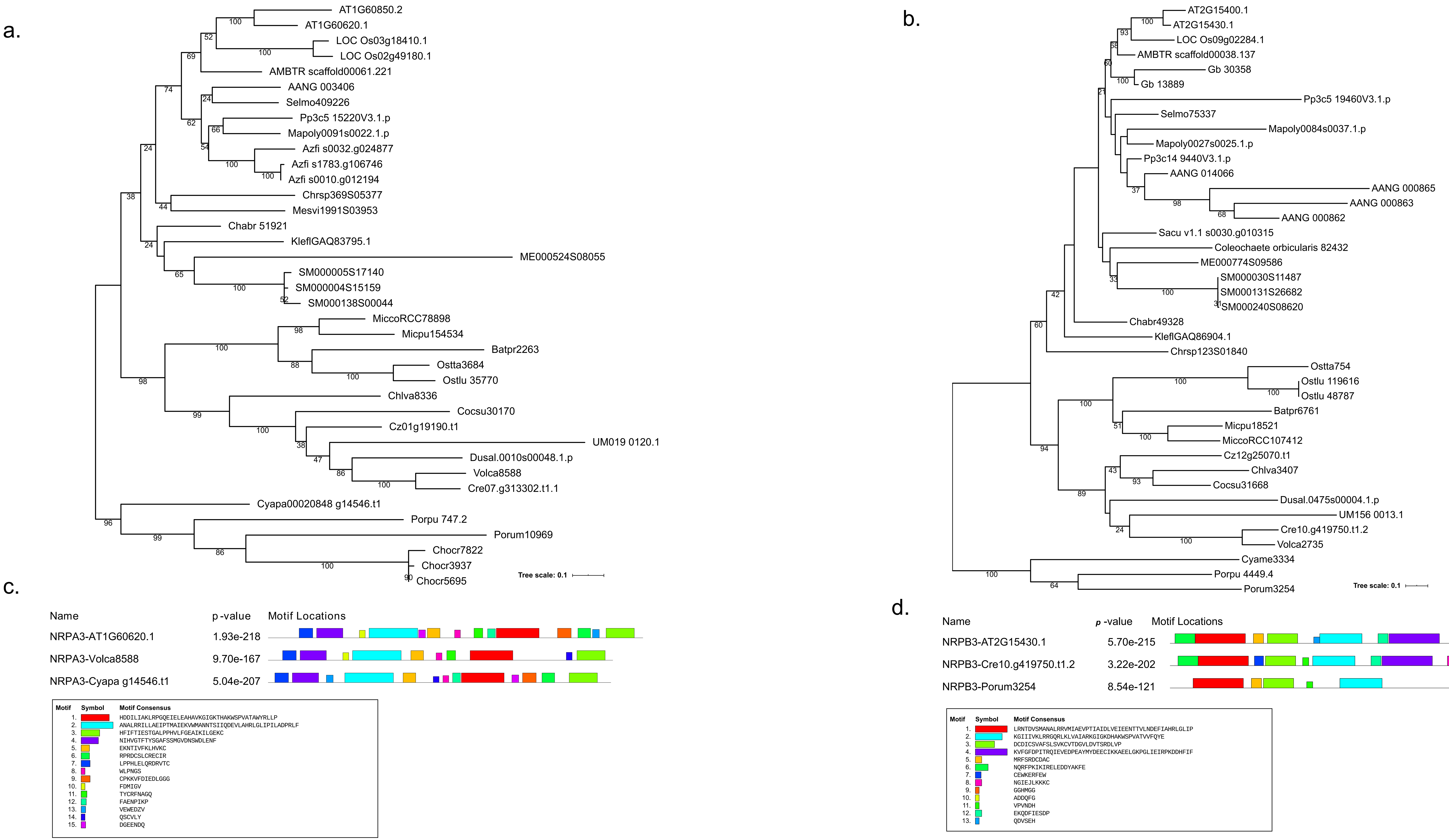

FigS16. The phylogenetic trees a of NRPA3 and NRPB3 genes in Archaeplastida

The phylogenetic trees and sequence motifs of NRPA3 and NRPB3 are shown here: (a). Maximum-likelihood tree of NRPA3 with 500 bootstrap replicates, generated by RAxML. The tree was rooted with Glaucoplantae and Rhodoplantae. (b). It's the phylogenetic tree of NRPA3 generated by RAxML with 500 bootstrap replicates. The tree was rooted with Glaucoplantae and RhodoplantaeBootstrap values are shown on each branch (0-100) and accession numbers to the sequences can be found in Supplementary data S3. (c) The motifs of NRPA3 are shown with 3 models and relatively complete sequences. (d). Motifs of 3 sequences of NRPB3 are shown. P-values motif locations are also presented.

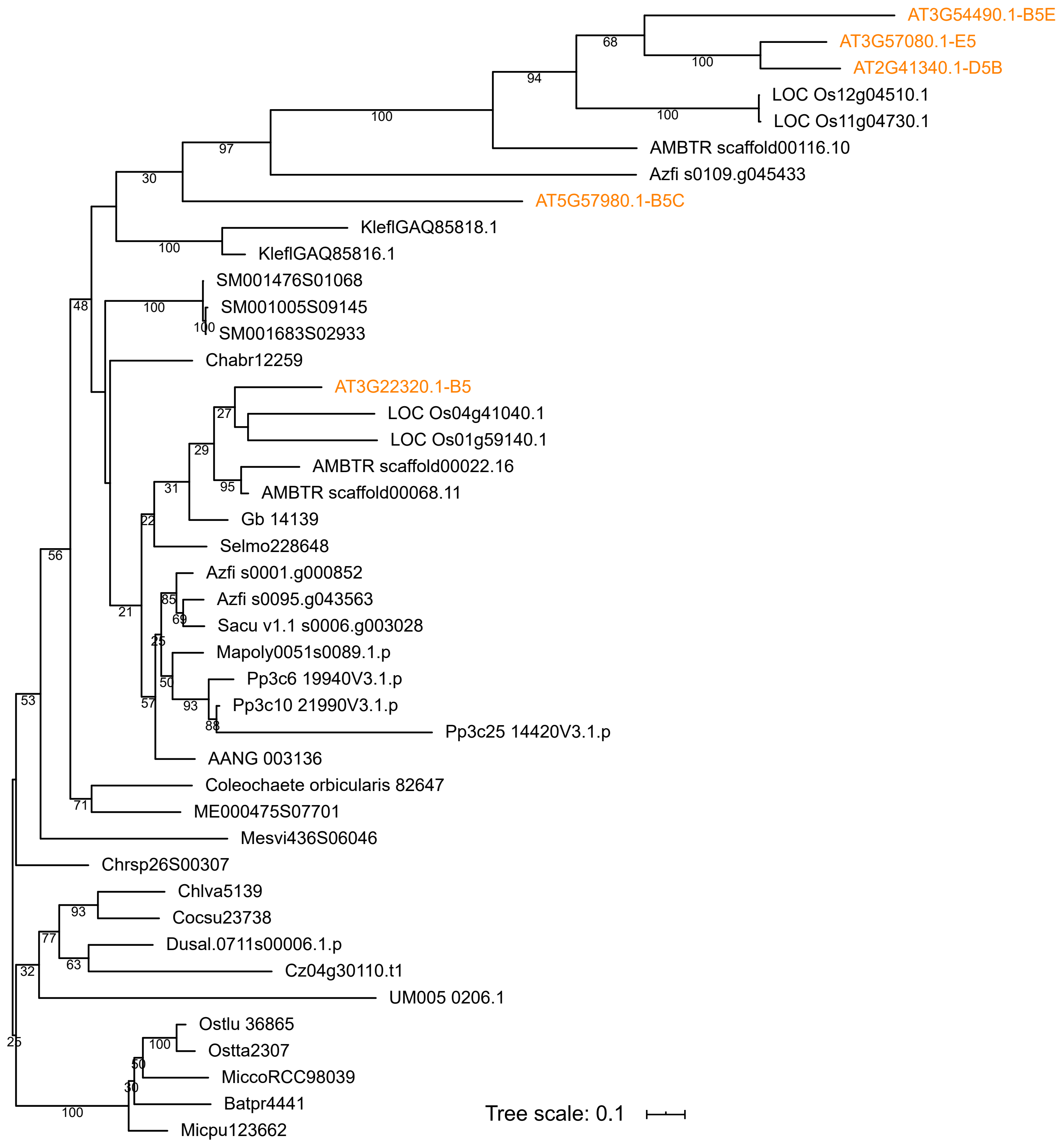

Fig. S17. The phylogenetic trees of NRPD5/E5

A Maximum-likelihood tree of NRPD5/E5 with 500 bootstrap replicates generated by RAxML with PROTCATGTR model.
