# Supplementary material for: Genome-wide analyses across Viridiplantae reveal the origin and diversification of small RNA pathway-related genes
Source: Commun Biol. 2021 Mar 25;4:412. doi: 10.1038/s42003-021-01933-5 (PMC7994812; doi:10.1038/s42003-021-01933-5)
Supplement: Supplementary file 3 — Description of Supplementary Files [file 42003_2021_1933_MOESM3_ESM.pdf]

Description of Additional Supplementary Files

File name: Supplemental Data 1-6
Description:

Supplementary data S1. Taxonomy and sequencing data information of the species used in this study.

Supplementary data S2: Detailed information regarding the species used in phylogenetic analyses.

Supplementary data S3: The genes involved in small RNA pathways (in this study).

Supplementary data S4: Description of domains in NRPD1/E1 protein.

Supplementary data S5: Topology comparison in different methods of all streptophytae algae branches in AGO tree.

Supplementary data S6: Models used in different phylogenetic trees.
